# Supplementary material for: Are carbon emissions trading and green financial instruments synergistic? -Comprehensive quantitative research based on content analysis
Source: PLoS One. 2024 Mar 7;19(3):e0298601. doi: 10.1371/journal.pone.0298601 (PMC10919601; doi:10.1371/journal.pone.0298601)
Supplement: S1 Data — (ZIP) [file pone.0298601.s001.zip › Data/地级市Z_F工作报告.zip!/║■▒▒/╛ú├┼╩╨/└┤╫╘╣1⁄2╓┌║┼í╛┬φ┐╦╩2╛▌═°í┐_2015.docx]

各位代表：

现在，我代表市人民政府向大会报告工作，请予审议，并请各位政协委员和列席人员提出意见。

2014年工作回顾

过去一年，面对经济增长速度换挡期、结构调整阵痛期、前期刺激政策消化期“三期”叠加的新形势，我们在省委、省政府和市委坚强领导下，在市人大、市政协监督支持下，认真贯彻落实党的十八大和十八届三中、四中全会精神，深入实施“两大战略”，致力“加速转型、缩差领跑”，较好地完成了全年工作目标任务。

——综合实力进一步增强。全市实现地区生产总值1310.59亿元，增长9.9%，高于全省平均水平0.2个百分点，增幅居全省第1位；规模以上工业增加值增长11.2%，居全省第2位；社会消费品零售总额451.78亿元，增长14.1%，增幅居全省第1位；财政总收入139.29亿元，增长13.5%，其中地方一般公共预算收入69.83亿元，增长20.3%。纳入全省县域经济考评的县（市、区），东宝位列一类第7名，钟祥、京山分列二类第3、4名，沙洋进位态势明显。

——经济结构进一步优化。全市三次产业结构比由15.8：54.2：30.0调整为15.1：53.9：31.0。完成高新技术产业增加值144.62亿元，增长15%；高新技术企业达到78家。强力推进节能减排，超额完成省定目标。

——项目投资进一步扩大。实现固定资产投资1190.15亿元，增长21.6%；实际外商直接投资29559万美元，增长12.5%；出口76749万美元，增长24.4%；实施亿元以上项目516个。

——人民生活进一步改善。全市民生投入146亿元，增长21.7%。2014年初承诺的民生实事全部办结。居民消费价格指数涨幅控制在1.9%以内；城镇登记失业率控制在4.3%以内；人口自然增长率4％。；城镇和农村居民人均可支配收入分别增长9.6%、11.6%。

一年来，我们主要做了以下工作：

（一）着力推动工业转型。大力调整优化工业结构，全市实施工业技改项目325个，投资169.4亿元，占工业投资的35.2%；淘汰水泥产能88万吨、平板玻璃产能85万重量箱、造纸产能2.5万吨。全国首个通用航空综合体——中航荆门爱飞客镇正式落户并获民航经营许可，省内首条低空训练和旅游航线开通；武汉国家生物产业基地荆门产业园获批；我市通过国家循环经济试点市验收，获批国家资源综合利用“双百工程”示范基地。院士专家工作站达到21个，国家电子废弃物循环利用工程技术研究中心在格林美挂牌。“一核六片十五园”实现工业总产值1659亿元，增长15.1%。荆门国家高新区辐射带动“一区四园”发展格局初步形成。深入开展工业经济“六比六看”竞赛和“进百企、解难题”活动，共设立5.6亿元调度资金，投入2.9亿元政府风险补偿铺底资金，清理处置低效闲置土地3550亩，推动“招工大篷车”下乡，开通“电力报装动车组”，全力支持企业生产经营。全市新增规模企业135家，达到1027家。培育工业化、信息化“两化融合”示范企业59家，凯龙进入国家级示范企业行列。

（二）着力夯实农业基础。中国农谷三大示范区建设扎实推进，国家农业科技园区创建工作通过专家考察，中兴农谷产业园奠基开工，农村综合产权交易中心挂牌，屈家岭农产品交易大市场（一期）主体工程完工，农业物联网生产管理平台、赵集智慧农业园智能控制系统投入运行，双碑农业示范园被评为全省十大设施蔬菜基地，石牌“1+5”生态文明新农村示范区、双龙苑等新型社区建设有序推进。大柴湖经济开发区规划体系基本形成，吉象贝儿童装等项目落户柴湖工业园区，移民生态新城开工建设。奋力“一抗三保”，战胜“五年连旱”，粮食总产达到54.59亿斤，实现“十一连增”。大力发展高效农业，推广农业新品种、新技术、新模式117项、面积180万亩。畜牧业发展水平进入全省前列，水产品产量增幅保持全省首位。农产品加工产值与农业总产值之比达到3.28：1，新增10亿元农业产业化龙头企业2家、“三品一标”企业176家，“龙池桥”被认定为中国驰名商标。主要农作物耕种收综合机械化水平达到75%，秸秆综合利用率达到83%。新型农业经营主体突破1.6万家。实施土地整治50万亩，城乡建设用地增减挂钩项目4200亩。完成沟渠清淤1996公里，扩挖改造塘堰5210口，新增和改善灌溉面积40万亩。屈家岭汉江引水工程全面启动。钟祥位居全省“三农”发展综合考评第一名，京山、沙洋进入前十强。

（三）着力增强内生动力。着眼“快增长、扩投资”，扎实开展项目建设、招商引资“两大竞赛”，全年新开工亿元以上项目245个，格林美东区、京峻汽车零部件、化工循环产业园管廊工程、牧原40万头生猪产业园等项目建设进展顺利，华能热电联产、台湾宝成工业园、渝楚化工异辛烷、明达LOW-E浮法玻璃等项目建成投产。荆门石化油品质量升级及适应性改造前期工作基本完成，进入环保部最后评审阶段。漳河机场改扩建可研报告通过评审；蒙华铁路即将开工建设，武荆城际铁路规划方案通过专家咨询论证；枣潜高速公路前期工作完成并与省交投公司签约，荆门城区绕城公路建设步伐加快，钟祥汉江二桥动工兴建，荆新线一级公路全线改造基本完成；沙洋港中心港区一期综合码头水工工程竣工，钟祥港石牌综合码头建设启动。编制完成荆门现代综合物流园规划，万达广场、多辉农产品物流中心、聚四海水产品批发市场、金瑞物流园等重点项目加快推进。举办油菜花旅游节、菊花展等特色旅游活动，我市荣获新华网“最美中国·人文休闲旅游目的地城市”称号，全年接待国内外游客2114万人次、实现旅游综合收入107亿元。新建和改造村级综合服务社150家，建成再生资源回收站点200个。新增总部企业3家，总数达到7家；财政总收入过亿元乡镇达到6个。开展柴湖“新农金”试点，建成77个“双基双赢”信贷工作室，湖北银行在我市设立分行，市中小企业担保公司提供担保贷款19亿元，全市各项贷款余额669.13亿元，比年初增加101.18亿元。新洋丰、雄韬股份成功上市，金源食品等11家企业在四板挂牌，市城投参股的光大幸福国际租赁有限公司在上海自贸区成立，上海股权托管交易中心荆门中小企业孵化基地落户。创办“荆门电子商务孵化中心”，入驻企业11家，东宝被列为全省首批电子商务进农村试点。

（四）着力打造美丽城乡。坚持城乡一体化与新型城镇化同步发展，全市城镇化率达到52.76%。《荆门市城市总体规划（2013-2030）》获省政府批准，鄂中城市群总体规划和建设行动方案编制完成，“数字荆门”地理空间框架建设与应用示范项目通过验收。漳河新区骨架初步拉开，漳河大道城区段基本完工，漳河国家湿地公园试点通过国家验收。竹皮河综合治理工程全面启动，汇合口浏河段和海慧沟段截污改造项目即将竣工。葛洲坝水泥异地技改项目开工建设，老城区生产线彻底关停。在全省率先启动中心城区全域集中供热项目，关停小锅炉160多台（套）。完成象山大道等35条道路维修工程，新增空调公交车100台、公共停车位300个。开展环境卫生、经营秩序、建筑渣土、车窗抛物、噪声油烟等专项治理，省级市容环境美好示范路创建稳步推进，违法建设得到有效遏制。各县（市、区）新区建设步伐加快，钟祥入选“2014中国最美30县”，京山成为全省首个省级生态县。钱场、后港、子陵、漳河、团林等10个镇入围全国重点镇，胡集进入省十强乡镇行列。建成155个新型农村社区。完成29座农村污水处理站建设，农村生活垃圾收运处理体系不断完善。强化土地矿产卫片执法，我市被列为全国工矿废弃地复垦利用试点市。基地造林15.6万亩，新建绿色通道654公里。建设二级公路139.56公里、县通乡公路247公里、通村公路686公里。

（五）着力深化各项改革。强化顶层设计，“6+N”重点改革事项全面启动。开展第9轮审批流程再造，市级行政审批事项缩减至126项，服务时限提速15%，零收费率达到95%，免收费用1.4亿元。借鉴上海自贸区经验，在荆门高新区成立行政审批局和综合执法局，实行“一枚印章管审批、一支队伍管执法”。京山建成全省首家县级公共检验检测中心。在9个乡镇开展公共资源交易试点。完成卫生计生机构改革和食品药品监管体制改革。实施事业单位分类改革，市直事业单位从355家整合为268家，核减编制980名。市直国有企业清产核资和分类工作有序推进。整合财政涉农资金25亿元。实行“工商新政”和企业登记注册“三证联办”，市场主体达到15.13万户。农村集体建设用地确权登记颁证完成60%，农村产权融资体制改革全面启动，新增流转土地55.19万亩，转移农民12.96万人。

（六）着力增进群众福祉。新增城镇就业5万人，扶持创业7000人，组织就业创业培训2.6万人，设立1000万元专项资金扶持高校毕业生创业就业。五大社会保险参保194.6万人次，城乡居民养老保险参保率达99.87%。在沙洋、钟祥开展公立医院改革试点，推广“先看病、后付费”服务模式；全面实施城乡居民大病保险工作；市一医南院、市精神卫生中心、市中医医院住院大楼主体工程竣工。在全省率先出台乡村医生生活补贴政策。新开工建设保障性住房、棚户区改造住房17164套，基本建成12792套，分配入住9687套，新增发放租赁补贴1190户。全市平价蔬菜直销店达到65家。城乡低保再次提标，发放医疗救助金2937万元、临时救助金2077万元。建成社区居家养老服务中心58个、农村老年人互助照料活动中心109个、青少年阳光家园18所。新建或改扩建幼儿园20所、留守儿童服务站10所，楚天学校建成投入使用，全域通过国家义务教育发展基本均衡县（市、区）评估，荆门技师学院被认定为“国家级重点技工院校”。新广播电视发射塔投入使用；新建村级体育健身工程208个；我市获省十四运会群体类金牌第三名。落实“单独两孩”政策，计划生育基层基础工作经验在全国交流。第三次经济普查有序开展。圆满完成第九届村委会换届。深入开展社会矛盾纠纷排查化解，办理法律援助等事项12770件。农村网格化建设在全省率先实现全覆盖。食品药品监管全面加强，安全生产形势总体平稳。完善社会治安动态防控体系，严密防范和严厉打击各类违法犯罪，群众的安全感、治安和执法满意度大幅提升。

各位代表，过去的一年，全市各条战线开拓创新，奋发有为。国防动员、人民防空、民兵预备役、双拥共建工作不断加强，工会、青少年、妇女儿童、老龄、残疾人等事业全面发展，市档案馆晋升国家二级档案馆，机关事务管理、新闻出版、移民、保密、仲裁、地方志、对台、气象、防震减灾、住房公积金、政府采购等在服务经济社会发展中发挥了积极作用，民族宗教、外事侨务、扶贫开发、援疆援恩等工作取得新成绩。

过去的一年，我们深入开展党的群众路线教育实践活动，着力转变职能、改进作风，政府自身建设进一步加强。全市行政事业单位“三公”经费下降35%，市政府会议和发文数量分别减少11%和20%。坚持依法行政，修订完善政府工作规则，出台重大行政决策程序规定、市级政府投资项目建设资金管理、政府合同管理等办法，全面清理规范性文件，聘请政府法律顾问，行政负责人出庭应诉率达100%，政府透明度排名全国地市级第4位。自觉接受监督，共办理人大建议109件、政协提案391件，满意率达98%以上。加强审计监督、行政监察和行风评议等工作，在全国率先推行财政大监督机制，举办3期电视问政活动。认真落实“一岗双责”，深入开展纠风治乱，严肃查处违法违纪案件，廉政建设和反腐败工作纵深推进。

各位代表，上述成绩的取得，是省委、省政府和市委正确领导的结果，是市人大、市政协监督和支持的结果，是全市上下团结一心、奋力拼搏的结果。在此，我代表市人民政府，向全市人民，向人大代表、政协委员，向各民主党派、无党派人士、工商联和人民团体，向驻荆部队、武警官兵和政法干警，向在荆中省单位和广大外来投资者，向所有关心、支持荆门发展的各界朋友，表示衷心的感谢并致以崇高的敬意!

在总结成绩的同时，我们也清醒地看到，我市经济社会发展中还存在一些突出问题。主要表现在：宏观环境错综复杂，经济下行压力较大，国内生产总值、规模以上工业增加值、固定资产投资增幅虽然“高于全省平均、同类城市领跑”，但与预期目标还有一定差距；经济总量不大和结构不优并存，“快增长”与“调结构”平衡难度增大；实体经济发展面临诸多困难挑战，融资难、融资贵和结构性缺工问题依然突出；民生工作还存在薄弱环节,公共服务资源配置不够均衡,城乡居民持续增收难度增加；政府职能转变有待进一步深入，发展环境有待进一步优化等。我们将采取更加有力的措施，认真加以解决。

2015年工作安排

2015年是全面完成“十二五”规划的收官之年，是全面深化改革的关键之年，是全面推进依法治市的开局之年，也是提前全面建成小康社会的攻坚之年。做好今年的工作，我们既要正视宏观经济下行压力，又要看到我市经济持续向好的态势已经形成；既要正视荆门发展不够的阶段性特征，又要看到转型升级是我市行稳致远的动力之源；既要正视财政实力、社保水平与民生需求的差距，又要牢牢守住民生底线，适应新常态，抢抓新机遇，干出新作为。

今年政府工作的总体要求是：全面贯彻落实党的十八大及十八届三中、四中全会精神，主动适应经济发展新常态，坚持稳中求进工作总基调，坚持市场、绿色、民生“三维纲要”，坚持“加速转型、缩差领跑”基本思路，深入实施“两大战略”，着力抓调整、促转型，抓投资、促增长，抓改革、促创新，抓民生、促和谐，全面完成“十二五”规划目标任务，为提前全面小康、建设“四个荆门”奠定坚实基础。

经济社会发展主要预期目标是：地区生产总值增长10%左右，规模以上工业增加值增长12%左右，全社会固定资产投资增长22%以上，社会消费品零售总额增长13.5%，实际外商直接投资增长10%，出口总额增长11%，地方一般公共预算收入增长13%以上，城镇常住居民人均可支配收入增长10%，农村常住居民人均可支配收入增长12%，城镇登记失业率控制在4.5%以内，居民消费价格涨幅控制在3.5%以内，人口自然增长率控制在5％。以内，节能减排完成省定目标。

重点抓好以下六个方面的工作：

（一）坚持优化结构，打造荆门工业经济“升级版”

以壮大总量、调整存量、做优增量为主攻方向，大力实施工业转型升级“四大工程”，进一步夯实工业主导地位。

实施传统产业提升工程。编制并落实传统产业转型升级“1+X”规划，推动石化产业向炼化一体化转变，磷化产业向食品级、电子级方向深化，建材产业向新型化、链条化迈进，机械产业向成套化、智能化跃升，纺织产业向规模化、精品化发展，农产品加工业向精细化、高端化转型，力争六大传统产业总产值突破2800亿元。推广沙洋玻璃产业转型经验，鼓励各类企业开展技术改造，突出抓好荆门石化油品质量升级及适应性改造、盈德煤制氢、沃裕化工硫酸钙晶须、葛洲坝水泥异地技改、楚商产业城、中兴农谷产业园等项目建设。坚定不移化解过剩产能，压减水泥产能195万吨、平板玻璃产能76万重量箱。

实施新兴产业培育工程。大力发展高端装备制造、节能环保、通用航空、新材料、电子信息、生物医药和健康等新兴产业，力争总产值突破300亿元。抓好中集化工能源装备、叶威智能物联网及粮食机械等项目，加快推进荆门高端装备制造产业园建设。用好国家节能减排财政政策综合示范市政策，依托国家资源综合利用“双百工程”和格林美国家“城市矿产”示范基地，实施鄂中再生资源大市场等50个循环经济项目，完成节能减排示范项目投资16.7亿元，积极创建国家循环经济示范市。以爱飞客镇建设为重点，做大做强荆门航空产业园，打造通用航空创意经济示范区。支持金泉新材料、智展光学、金龙非、金汉江等企业抢占发展制高点，培育新材料产业集群。推进华工科技荆门光电子信息产业园、鸿创科技等项目建设，力争尽快形成产能。依托武汉国家生物产业基地荆门产业园，积极发展生物医药、农用生物制品、生物能源等特色产业，支持神地、欣胱、润红等企业扩规增容。

实施中小企业成长工程。选择一批成长性好的企业进行重点培育，引导食品、建材、机械、磷化等行业的小微企业联合壮大，确保新增规模以上工业企业100家以上。鼓励龙头企业外引内联、做大做强，力争亿元以上企业新增75家，推动新洋丰、京山轻机、国宝桥米、洪森等向百亿企业进军。引导企业完善法人治理结构和现代企业制度，培育一批具有国际眼光的优秀企业家。深入推进质量兴市、品牌强市战略，选择200家创新能力强、发展前景好的中小企业争创精品名牌。

实施工业园区增效工程。以扩园强园为抓手，深化“一核六片十五园”建设。对符合条件的省级开发区进行扩区调区，努力在功能配套、产业聚集、环境提升等方面取得新进展。建立健全入园项目“六度”考评体系，支持各园区做大做强优势产业，力争全市园区实施亿元以上项目360个，总产值增长25%。发挥荆门国家高新区体制机制优势，通过扩大管辖权、下放审批权、整合执法权、赋予人事权和优化财税权，扎实推进“一区四园”建设。支持沙洋经济开发区创建省级高新区。

（二）坚持强农富农，深入推进国家现代农业示范区建设

加快转变农业发展方式，全面提升农业集约化、专业化、组织化、社会化水平，使农业强起来、农民富起来、农村美起来，在全省率先基本实现农业现代化。

强化示范引领。持续推进中国农谷三大示范区建设，打造“三农特区”。在农谷核心区，加快实施联想高档水果产业园、万亩玫瑰产业园等项目，依托中兴、汇源等企业发展智慧农业，建成院士村一期项目，创建国家农业科技园区；在“荆三角”，提升五洋公路油菜花景观生态农业旅游线档次，推进凯跃农机博览城建设；在杨竹流域，抓好石牌“1+5”示范区、双龙苑万人社区建设，力争汉光农业物流园、富硒米业等项目尽快投产见效。大力推进大柴湖开发区建设，深化柴湖工业园与武汉东湖高新区结对共建，扶持罗成村整村推进土地流转和发展高效农业，加快移民生态新城建设步伐，完成1000户危房改造任务。

大力调整农业结构。围绕“一高三新”和“一抗三保”，进一步优化农业种养结构，积极推广“香稻嘉鱼”“42养羊”等27种生态种养模式，力争主推优质品种普及率提高到75%以上。继续开展粮食稳定增产行动和高产创建活动，突出抓好100万亩小麦、250万亩优质水稻、160万亩双低油菜核心示范区建设，力争粮食增产1亿斤。实施“菜篮子”工程，确保全市最低菜地保有量50万亩。推进“退耕还草”“种草养畜”项目，新建畜禽标准化养殖示范场80个；发展特色水产，高标准建好一批现代渔业示范园区。深入实施“四个一批”工程，打造一批自主创新能力强、加工水平高、处于行业领先地位的产业化龙头企业，农产品加工值与农业总产值之比达到3.3：1以上，新认证有机、绿色、无公害食品30个以上。

完善新型农业经营体系。培育壮大新型农业经营主体，力争总数突破2万家，其中农民专业合作社4400家、家庭农场780家。加强气象预警防灾、疫病防控、森林防火及农业政策性保险等工作，推行统一供种、统一育秧、统一植保、统一肥水管理、统一农机作业等专业化服务，建设农机化综合示范区。创新“三资”管理模式，发展壮大村级集体经济。完成农村集体建设用地确权登记颁证工作。落实农村集体土地所有权、承包权、经营权“三权分置”政策，推进土地承包经营权有序流转。深化农村产权融资改革，力争农村土地经营权和集体建设用地使用权抵押贷款规模达到2亿元。

改善农村生产生活条件。加快屈家岭汉江引水、荆门汉江以西水系连通及城市备用水源工程建设，完成42座小（2）型病险水库除险加固任务，加固汉江堤防17公里。拉通乡村断头路300公里，健全农村公路养护管理机制。新增清洁能源入户1万户，户用沼气使用率提高3-5个百分点。坚持最严格耕地保护制度，实施土地整治项目36万亩、城乡建设用地增减挂钩项目3000亩、工矿废弃地复垦项目5000亩。

（三）坚持提速提质，保持经济“快增长”态势

切实强化扩投资、促消费、强实体、增效益的各项措施，积极应对经济下行压力，努力实现有质量的“快增长”。

高位推进项目建设。把“扩投资”作为“快增长”的关键，持续推进项目建设、招商引资“两大竞赛”，全年亿元以上项目完成投资850亿元。健全重点项目推进机制，完善领导挂点包保、部门对口包联制度，开展项目拉练活动，促进项目快落地、快建设、快投产。大力推行政府购买服务和PPP等投融资模式，鼓励引导民间资本投向支柱产业、基础设施、民生改善等领域。加大项目策划包装、宣传推介、向上争取力度，力争普惠性项目获得更多份额、个性化项目取得更大突破。

大力发展现代服务业。促进制造业、商贸流通业与电子商务深度融合，做大做强电子商务园区，建成淘宝网荆门（中国农谷）馆，鼓励和扶持一批优秀电子商务企业发展壮大。支持东方百货、金城集团重组成立供销集团，打造百亿商贸航母。加快推进武商荆门广场、星球·世界城、一马光彩大市场等项目和各县（市、区）现代物流产业园建设。组建文体产业集团，提升文化产业发展水平。促进大洪山风景名胜区、纪南-纪山楚文化旅游景区、大太子（大口、虎爪山、太子山）森林休闲养生区资源整合，实施屈家岭考古遗址公园、汇源生态产业园、太子森林小镇等重点旅游项目。支持绿林山、明显陵创建5A级景区，漳河创建4A级景区，马河创建全省旅游名镇，积极筹备2016年中国（荆门）菊花展，办好第八届油菜花旅游节及首届紫薇花旅游节、首届中航爱飞客通用航空大会和中国（京山）第二届网球节，力争全年接待游客2500万人次，实现旅游综合收入130亿元。培育规范房地产市场，促进房地产业持续健康发展。加快“智慧荆门”建设，大力发展健康服务业，不断扩大保险、家政、文化创意等热点消费，推进特色商业街和夜市建设，确保服务业发展提速、比重提高、水平提升。

强化财税金融工作。以总部经济、支柱产业为重点，统筹推进市、县、乡财源建设，力争新引进总部企业5个左右，财政收入过亿元的乡镇达到8个。加强税收征管，促进财政收入稳定增长。深化预算管理制度改革，加强政府性债务管理。引导金融机构创新金融产品和服务，开展县域金融工程试点，加快创建普惠金融和信用建设示范区，加强对小额贷款公司、融资性担保企业的监管和引导，力争新增贷款100亿元以上。强化金融信用体系建设，大力防控金融风险，优化金融生态环境。建立荆门中科创新创业投资基金，创新科技投融资机制。支持凯龙加快上市步伐，力争一批企业分别在三板、四板和上海股权交易中心挂牌。

加强对实体经济的支持。落实中小微企业发展扶持政策，深化“进百企、解难题”活动，建立中介服务超市，完善中小企业服务中心功能。发挥财政资金杠杆作用，探索建立产业发展基金，市财政投入资金3亿元支持企业发展。加强土地资源统筹配置，继续清理处置低效闲置土地，确保重点项目建设用地。加强校企合作、定向培训和村企对接，实行“一对一”“点对点”招工，破解企业“用工难”问题。严控各类涉企检查，坚决取消各种不合规收费，不断优化经济发展环境。

（四）坚持城乡一体，统筹推进新型城镇化

紧紧围绕产城融合、“四化”同步，大力实施鄂中城市群总体规划和建设行动方案，力争新城新形象、老城有新样、镇村变模样。

做精做优中心城区。坚持新区建设和老城改造同步推进，打好中心城区升级攻坚战。全面完成漳河新区规划体系，扎实推进双喜大道、荆山大道等道路建设，确保漳河大道全线通车；加快实施万达广场、红星国际广场、国华人寿等项目，打造现代服务业聚集区；建成凤凰湖湿地公园，创建国家绿色生态示范城。统筹抓好东宝长宁新城、掇刀长坂新城、荆门高新区观堰湖生态科技新城建设，提升麻城、团林、漳河、子陵和牌楼“五组团”建设水平。进一步提升老城区功能，积极推进葛洲坝水泥老厂区等棚户区改造，加快城区冷热电三联供建设进度，综合整治杨家桥等高速公路出入口环境，配套完善城市基础设施，新建一批小游园、小广场，让城市更靓丽、生活更美好。

构建多点支撑格局。支持京山温泉新区、沙洋滨江新区和新港区、钟祥莫愁湖生态新区建设，推动县城建设上水平。以扩权强镇为重点，促进宋河、钱场、后港、胡集、柴湖、易家岭6个小城市加快发展，抓好官垱“四化”同步省级示范镇试点工作。坚持全域规划、镇村联动，积极推进孙桥、牌楼等5个省级新农村试点镇和42个省级新农村示范村建设，完成第二批100个新型农村社区详规编制。抢抓江汉运河生态文化旅游城镇带建设机遇，打造“灵秀毛李”等魅力水乡小镇。支持各县（市、区）因地制宜、竞相发展，比学赶超、保强进位，进一步增强县域实力。

提高城市管理精细化水平。巩固城管“三年行动计划”成果，推动城市管理向精细化、智能化转变。力争背街小巷、城乡接合部和城郊标准化人工保洁全覆盖，道路机械化洗扫作业覆盖率达到80%以上。重拳整治交通违规违法行为，完善道路微循环系统，整顿出租车市场，加强占道经营监管，新建立体式停车场，切实缓解中心城区交通拥堵问题。加大控违查违力度，确保无新增违法建设，存量违建逐年减少。强化电子效能监察，拓展数字城管平台功能。加强对县（市、区）城市环境综合整治考评，全面提升城市品位和形象。

全力推动城乡互通互联。围绕构建“三环八射六隧”城市路网，加快中心城区绕城公路建设步伐。力促枣潜高速公路荆门段开工建设，钟祥汉江二桥主体工程加速推进。全力服务蒙华铁路建设；深化武荆城际铁路前期工作，力争早日开工。抓好石牌港区建设，确保沙洋港区综合码头试运营，推进碾盘山水利枢纽等工程前期工作。积极实施漳河机场改扩建项目。全面完成一、二级公路和县通乡三级公路年度建设任务。围绕构建“零距离换乘”体系，加强铁路、公路、城市公交站场规划建设。

（五）坚持创新驱动，激发发展活力

全力推进改革攻坚、市场开放，聚合正能量、释放新红利，让一切创造社会财富的源泉充分涌流。

抓好改革措施落地。深化行政审批制度改革，减少前置审批，优化审批流程，加强事中事后监管，擦亮“四最四零”品牌。以行政审批、综合执法、科技创新、科技金融和扩大开放为重点，推动“一区四园”先行先试，构建政策共享机制。推广京山整合组建公共检验检测平台经验。基本完成市直事业单位分类改革和全市公务用车制度改革。抓好中介机构脱钩改制。引导协会、商会等社会组织健康发展。以管资本为主，理顺国有资产监督管理体制机制，提高国有资本运营效益。拓展公共资源交易范围，抓好电子化招投标工作。加快户籍制度改革，有序推进农业转移人口市民化，力争城镇化率提高2-3个百分点。

扩大对外开放与合作。坚持产业招商、以商招商、“点球”招商，加快引进产业龙头项目和产业链节点项目。加强重点出口基地建设，扩大机电、纺织服装、优质农产品和服务贸易出口比例，促进外贸出口多元发展。支持格林美申报B型保税物流仓库。鼓励京山轻机、凯龙化工、新洋丰等优势企业“走出去”，开拓国际市场。积极融入“两圈两带”，在汉江生态经济带开放开发中唱好“重头戏”。

促进大众创新创业。实施市场主体增量行动，以招商促创业、创业带就业。发挥聚盛科技孵化器、百盟加速器及荆门台银两岸三地华中创业中心功能，厚植“草根经济”发展土壤，新增市场主体1.2万家以上。依托“院士专家工作站”，促进一批先进适用技术就地转化应用。实施“科技创业带头人扶持计划”，开展“千名科技精英进荆门进千企”“院士专家荆门行”活动，发挥格林美国家级工程技术研究中心作用，力争新增高新技术企业30家以上、省级以上研发中心2-3家，全市高新技术产业增加值增长15%以上。大力实施产业人才“金蓝领”工程，引进30名左右高端创新创业领军人才，培育高技能人才800人以上。加强专利运用和保护，力争全市专利授权总量超过580件。

（六）坚持民生优先，推动发展成果共享

着力构建普惠、均衡、优质的公共服务体系，让所有需要帮助的人们生活得到保障、心灵充满温暖，让全市人民共享美好生活。

加强就业和社会保障工作。创新招工、培训、就业“三位一体”机制，统筹做好高校毕业生、农村转移劳动力、城镇困难人员、退役军人等群体就业工作。开展全民参保登记试点，五大社会保险参保达到196万人次。推进基本养老保险城乡统筹，提高新农合筹资、城乡居民大病保险水平。强化城乡居民最低生活保障，加大社会救助力度，建立“一门受理、协同办理”机制，重特大疾病住院医疗救助比例提高到65%。加快市残疾人康复中心建设。完善住房公积金制度，健全住房保障和供应体系。推进移民后扶工作。实施精准扶贫，减贫4万人。加强职工服务站点建设，抓好农民工工资清欠工作，维护劳动者合法权益。

大力发展社会事业。广泛开展科普宣传教育，提升公民科学素养。加大学前教育普及普惠力度；优化城乡义务教育资源配置；实施普通高中质量提升和特色发展工程；积极发展现代职业教育和特殊教育；支持荆楚理工学院向应用技术型大学转型。加强学校文化建设，全面推进素质教育。开工建设望兵石学校。力争青少年综合实践基地年内投入使用。推进“三中心”、市民中心、市博物馆新馆及县（市、区）“三馆”建设，建好一批乡镇文化综合体项目。实施全民健身工程，免费开展公益艺术体育培训，办好市第八届老年人健身大会、社区文化节等群众性文化活动。积极发展哲学社会科学，鼓励文艺精品创作。完成农村智能广播网建设，推进广播电视直播卫星户户通工程。深化医药卫生体制改革，推动县级公立医院综合改革全覆盖，开展“分级诊疗”试点，推行基本药物制度进社区卫生服务站，按政策落实村医待遇。稳定低生育水平，促进性别比均衡发展，启动计生奖励政策全覆盖工作。大力实施妇女儿童发展规划，切实保障未成年人合法权益。加强国防动员、民兵预备役和人民防空等工作，抓好全民国防教育，促进军民深度融合。协调发展民族宗教、外事侨务、新闻出版、工会、老龄、对台、保密、档案、地方志、防震减灾、援疆援恩等各项社会事业。

加快生态家园建设。坚持国家生态市、环保模范城市、卫生城市和森林城市“四城同创”，推动绿色发展。持续推进“向污染宣战三大行动”，坚决落实漳河三圈保护规划，加强长湖水环境综合治理，大力防治工业、扬尘、机动车尾气和餐饮业油烟污染，做好秸秆禁烧和综合利用工作。抓好矿山地质环境工程治理。深化“绿满荆门、花开农谷”行动，加强森林资源保护，完成基地造林12.5万亩、绿化通道400公里，打造秋游农谷紫薇花海景观带。深入开展美丽乡村建设，推进村庄环境综合整治，减少农村面源污染，支持130个村创建省级绿色示范村。

加强和创新社会治理。深化法制宣传教育和依法治市工作，创建全国法治城市。进一步完善人民调解、行政调解、司法调解联动机制，大力化解信访积案。完成社区建设“三年行动计划”任务，抓好第六届社区居委会换届选举。加强城乡社区网格化管理，推进便民服务下沉。加强安全生产隐患排查治理，坚决遏制重特大安全事故发生。强化食品药品质量安全监管，开工建设荆门市食品药品检验检测中心。落实“三全巡逻”“三边警务”和单位内保制度，持续开展严打整治，深化“平安荆门”创建。

各位代表，民之所望，施政所向。今年我们将重点办好以下民生实事：

1.城镇新增就业4.5万人，组织城乡劳动者就业培训1.9万人，扶持创业5500人，带动就业2.2万人。

2.实施竹皮河城区段清淤筑坝、生态补水、景观改造、设施配套和面（点）源治理工程，完成浏河示范段建设。抓好象山大道、白云大道等道路美化、亮化工作，打造1-2条省级市容环境美好示范路。新建4座垃圾转运站。

3.对市特殊教育学校学生实行“全免”政策；资助100名荆门籍贫困大学新生。免费为1万对育龄群众开展孕前优生健康检查，为200例贫困白内障患者实施复明手术。

4.拉通白庙路、果园一路、莲花路、长莱巷等影响交通畅通的断头路、瓶颈路；新增100台空调公交车。

5.新建10个省级示范宜居村庄；完成3000户农村危房改造。清淤扩挖塘堰5000口，清淤沟渠1500公里，衬砌农渠500公里。解决8万人的饮水安全问题。

6.举办惠民演出110场。对100处城区市民室外阅报栏进行扩规升级。

7.全市新开工建设保障性住房9368套，基本建成9746套，分配入住4585套，新增发放租赁补贴300户。

8.新建城市社区居家养老服务中心40个、农村老年人互助照料中心200个、留守儿童服务站10所。在中心城区新建青少年“阳光家园”10所。

9.按照每村100万元标准，支持23个重点老区村、贫困村发展。

10.为1万名贫困残疾人发放生活补贴，为1300名残疾人发放机动车燃油补贴，免费为贫困残疾人适配1000件辅助器具；为困难群众、弱势群体和农民工提供法律援助。

各位代表，政府的一切权力来自人民、源自法授。我们将依法全面履行政府职能，加快建设法治政府和服务型政府。

——切实做到“职能科学、权责法定”。积极稳妥地抓好政府机构改革，认真落实政府及其工作部门权力清单制度，推进机构、职能、权责、程序法定化。自觉接受人大及其常委会的法律监督、工作监督和政协的民主监督，认真办理人大议案、代表建议和政协委员提案，主动接受社会和舆论监督。

——切实做到“执法严明、公开公正”。始终运用法治思维和法治方式履行职能，完善政府常务会学法、政府法律顾问、规范性文件制定及备案等制度，健全重大行政决策程序，加强行政复议，坚持规范公正文明执法。深入开展损害群众利益突出问题专项治理，坚决纠正部门和行业的不正之风。加强电子政务和政府网站建设，强化舆情引导，推进信息公开。

——切实做到“廉洁高效、守法诚信”。认真落实政府党组党风廉政建设分主体责任，严格执行政治纪律、组织纪律、财经纪律，深入推进廉政风险防控管理，加强行政监察和审计监督，确保廉洁从政。厉行节约、反对浪费，严控“三公”经费。推动诚信体系建设，以政务诚信带动商务诚信和社会诚信。

——切实做到“以人为本、执政为民”。巩固党的群众路线教育实践活动成果，大力推行直接联系和服务群众制度，以“钉钉子”精神落实市政府5个方面、40项整改任务，构建反“四风”、转作风长效机制。加大“庸懒散软奢”整治力度，持续推进作风效能建设。按照“清廉为官、事业有为”要求，强化履职担当，对确定的目标、制定的政策、部署的任务，一抓到底，务求实效。我们一定牢固树立宗旨观念，通过一件件小事的办理、一件件实事的落实、一件件难事的解决，把群众的期待变成我们的行动，把群众的希望变成生活的现实。

编制“十三五”规划是今年的一项重要任务。我们将全面分析未来五年荆门经济社会发展面临的形势，集思广益、群策群力，切实做好规划编制工作，使之成为科学指导全市经济社会发展的宏伟蓝图和行动纲领。

各位代表！梦想承载希望，实干开创未来。让我们紧密团结在以习近平同志为总书记的党中央周围，在省委、省政府和市委的坚强领导下，紧紧依靠全市人民，解放思想，锐意进取，真抓实干，共同谱写实力、文化、生态、幸福荆门建设的新篇章！
